# Local mushroom poisoning: a case report study

**DOI:** 10.1093/omcr/omaf121

**Published:** 2025-09-15

**Authors:** Mahsa Rizebandi, Faezeh Sehtpour, Farhad Mohammadi

**Affiliations:** Assistant Professor of Internal Medician, Department of Internal Medician, Razi Hospital, Ilam University Sciences, Ilam, Iran; Endocrinology and Metabolism Research Center, Endocrinology and Metabolism Clinical Sciences Institute, Tehran University of Medical Sciences, Tehran, Iran; Graduate of Master of Biostatistics Employee of Razi Hospital, Ilam, Iran

**Keywords:** poisoning, local mushroom

## Abstract

Introduction: Poisoning by poisonous local mushrooms that resemble edible mushrooms is a serious health hazard with severe consequences. Wild and unidentified mushrooms are the main cause of these poisonings. Successful treatment requires prompt medical intervention, and if delayed, a liver transplant may be necessary. Case report: A 26-year-old woman with postpartum cardiomyopathy was admitted to Razi Hospital with abdominal pain, dizziness, nausea, and vomiting. She was diagnosed with poisoning from local mountain mushrooms and transferred to the ICU due to low blood pressure, elevated enzymes, and tachycardia. Echocardiography showed severe cardiac dysfunction (EF = 25%), and she developed severe tachycardia, decreased consciousness, and metabolic-respiratory acidosis. After dialysis and treatment with NAC infusion for 7 days, her consciousness improved, she was extubated, and EF increased to 40%. Conclusion: The patient gradually improved after 6 days in ICU, with pancreatitis resolving and bilateral lower extremity paresthesia diagnosed and subsequently improved.

## Introduction

Mushrooms are a fleshy fruit and food source, usually growing above ground in the soi [1]. The growing consumption of mushrooms has led to more poisoning cases due to misidentification of toxic species and improper cooking [2]. Mushroom poisoning symptoms vary by type and amount consumed, with Amanita species being the most dangerous [3]. Hypotension is the most common cardiovascular sign in mushroom poisoning. Other cardiac toxicities like arrhythmias, myocarditis, and ischemia have been reported, though the direct effects on the heart remain unclear [4]. In March 2024, a case study in China reported poisoning from wild mushrooms containing amatoxin affecting three people. All experienced gastroenteritis symptoms within 10–15 hours after ingestion, including vomiting, abdominal pain, and diarrhea. One individual sought prompt medical care and recovered successfully. However, the other two delayed treatment by two to three days, leading to worsening conditions and death [5]. Acute pancreatitis is a rare but often misdiagnosed complication of mushroom poisoning due to symptom similarity with mild cases. Early recognition and prompt intervention and treatment are needed to avoid severe complications in severe cases. Here is a contained list of symptoms caused by poisonous mushrooms, organized by body systems and ranked from most common to least common symptoms. This list is based on the type of toxin and the mushrooms involved.

**Figure 1:**
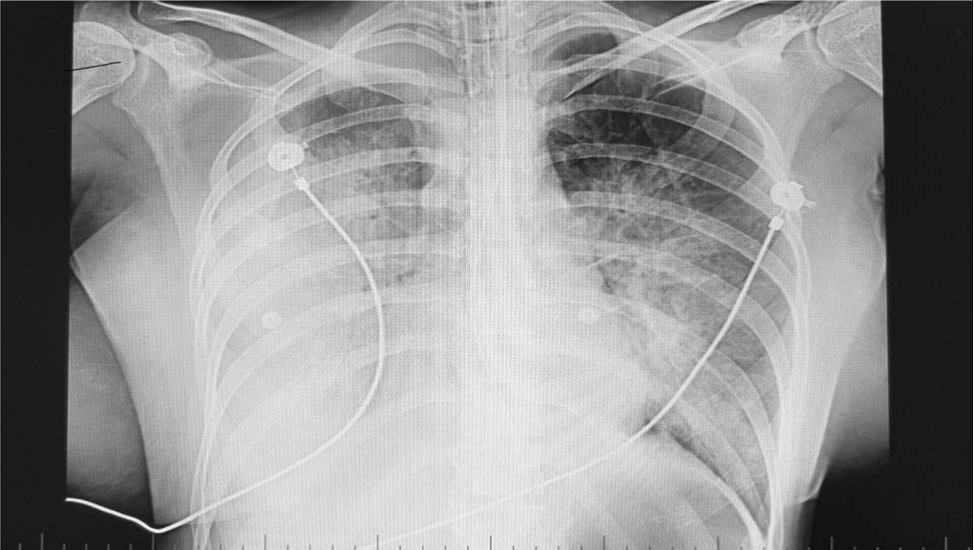
Chest x-ray finding on the third day of admission.

## Case report

In the present study, we report a 26-year-old woman with a medical history of postpartum cardiomyopathy (8 months ago) due to triplet pregnancy, who presented with abdominal pain, dizziness, nausea, and vomiting with a diagnosis of acute decompensated heart failure versus myocarditis and pancreatitis post mushroom consumption. In the initial assessment of the patient, his heart rate was 120, blood pressure 80/50 mm Hg, and body temperature 36.8. For this reason, the patient was transferred to the intensive care unit (ICU) for continuous monitoring of vital signs and clinical status. Despite initial treatment and hydration, the patient's tachycardia did not improve. 24 hours after admission, the heart rate reached 180, when the patient suddenly lost consciousness and evidence of wide complex tachycardia, ventricular tachycardia was observed. Given the simultaneous low blood pressure and the opinion of a cardiologist, he was treated with a 200 joule cardiac defibrillator shock (one time). The patient was intubated by the anesthesia team and the next day's echocardiography reported an ejection fraction of 25%.Lactate dehydrogenase, creatine phosphokinase, amylase, and lips were high; it is controversial whether these laboratory tests indicated pancreatitis because of N-acetylcysteine (NAC) or mushroom poisoning. It is not clear to us. She gradually became good and was transferred to a ward and her vital signs were good, after treatment of pancreatitis amylase and lips decreased, and after that, we detected bilateral lower limb paresthesia, and then after several days, she got better. Finally, she was discharged with good general conditions and it was recommended to follow up with the heart, neurology, and gastroenterology clinic. Here’s a short list of poisonous and non-poisonous mushrooms in tabular1 form.

**Table 1:** A list of symptoms caused by poisonous mushrooms, with the most common symptoms to the least severe.

System	Most Common Symptoms	Less Common Symptoms
Gastrointestinal	Nausea, vomiting, diarrhea, abdominal pain	Dehydration, bloody stools
Hepatic (Liver)	Jaundice, elevated liver enzymes	Hepatomegaly, hepatic coma
Renal (Kidney)	Oliguria, hematuria	Acute kidney injury, renal failure
Neurological	Hallucinations, confusion, dizziness	Seizures, coma, ataxia
Cardiovascular	Tachycardia, hypotension	Arrhythmias, cardiac arrest
Musculoskeletal	Muscle weakness, myalgia	Rhabdomyolysis
Dermatological	Sweating, flushing	Rash
Metabolic	Hypoglycemia, electrolyte imbalances	Metabolic acidosis
Respiratory	Hyperventilation	Respiratory failure

**Table 2:** Poisonous and non-poisonous mushrooms.

Poisonous Mushrooms	Non-Poisonous Mushrooms
Death Cap (*Amanita phalloides*)	Button Mushroom (*Agaricus bisporus*)
Destroying Angel (*Amanita virosa*)	Shiitake (*Lentinula edodes*)
Fool’s Mushroom (*Amanita verna*)	Oyster Mushroom (*Pleurotus ostreatus*)
Panther Cap (*Amanita pantherina*)	Morel (*Morchella spp.*)
Deadly Webcap (*Cortinarius rubellus*)	Chanterelle (*Cantharellus cibarius*)
Autumn Skullcap (*Galerina marginata*)	Porcini (*Boletus edulis*)

**Table:** Results of the treatment period:

	1^th^day	2^th^day	3^th^day	4^th^ day	5^th^day	6^th^day	7^th^day	8^th^day	9^th^ day
WBC(x1000/mm^2^)	10.3	-	19.3	13.6	13.8	10.2	12.5	8.7	-
HB(g/dl)	14.3	-	17.0	16.4	12.1	12.9	13.9	13.8	-
HCT%	36.3	-	51	44.8	36.0	37.4	39.9	37.4	-
MCV(fL)	87.6	-	90.0	86.5	89.0	86.0	86.9	87.0	-
PLT(x1000/mm^2^)	254	-	158	138	125	151	215	224	-
Urea(mg/dl)	38	20	34.0	35	33.0	26	22	16	18
CR(mg/dL)	0.9	1.4	1.4	1.8	1.2	1.0	0.9	0.8	0.8
Na(meq/l)	142	142	140.8	140.0	138.8	144	138	137.0	138
K(meq/l)	4.7	4.4	4.0	3.6	3.7	3.9	4.2	3.9	4.1
PT	16	-	25	-	15	15	-	-	15
PTT	26	-	75	-	30	44	-	-	30
INR	1.08	-	1.86	-	1	1	-	-	1
AST(U/l)	17	-	114	98	100	74	73	28	23
ALT(U/l)	10	-	68	73	187	154	103	103	76
ALK.P(U/l)	81	-	109	75	96	95	97	108	97
ALB(g/L)	3.9	3	-	3.9	-	-	-	-	3.4
T.BILI(mg/dl)	2.1	-	0.6	0.28	1.2	1.0	1.1	1.1	1.1
D.BILI(mg/dl)	0.4	-	0.2	0.15	0.3	0.4	0.3	0.2	0.2
PH	7.44	-	6.9	7.31	7.44	7.44	7.45	-	-
PCO2(mmHg)	32.0	-	82.6	47.6	33.0	30.1	36.5	-	-
HCO3(mmol/l)	22.0	-	15.8	24.0	22.2	20.6	25.3	-	-
Ca (mg/dl)	9.0	8	-	-	-	-	-	8.0	8.8
Pho (mg/dl)	1.2	2.8	1	-	-	-	-	3.1	3.6
Mg (mg/dl)	1.7	1.5	1.9	1.4	-	-	-	1.9	1.8
Amylase (U/l)	64	-	197	324	215	445	435	373	278
Lipase (U/l)	36	-	106	124	284	2880	2660	950	550
BS (mg/dl)	126	155	170	146	96	112	176	136	141
TG(mg/Dl)	-	-	-	-	-	119	-	-	-
LDL(mg/dl)	-	-	-	-	-	81	-	-	-
HDL(mg/dl)	-	-	-	-	-	27	-	-	-
Troponin	negative	negative	positive	-	-	-	-	-	-
CPK	261	191	1917	906	712	603	401	147	-
LDH	149	618	578	431	379	386	355	302	-
Retic%	-	0.8	-	-	-	-	-	-	-
Blood Culture	-	-	negative	-	-	-	-	-	-
Urine Culture	-	-	negative	-	-	-	-	-	-
COVID19 PCR	-	-	negative	-	-	-	-	-	-

Abbreviations: WBC, white blood cell; HB, hemoglobin; MCV, mean corpuscular volume; PLT, platelet; Na, sodium; K, potassium; PT, prothrombin time; PTT, partial thromboplastin time; INR, international normalized ratio; AST, aspartate aminotransferase; ALT, alanine aminotransferase; ALK.P, alkaline phosphates; ALB, albumin; T.BILI, total bilirubin; D.BILI, direct bilirubin; Ca, calcium; Pho, phosphor; Mg, magnesium; BS, blood sugar; TG, triglyceride; LDL, low-density lipoprotein; HDL, high-density lipoprotein; CPK, creatine phosphokinase; LDH, lactate dehydrogenase; PCR, *polymerase chain reaction*.

## Discussion

Mushroom poisoning poses a major public health risk, especially in regions with common wild mushroom foraging like Ilam Province, Iran. The case report highlights dangers from misidentification due to gaps between traditional knowledge and scientific mycology. Addressing these knowledge gaps is crucial to prevent future poisoning incidentsMany foragers mistakenly rely on visual cues like color and shape, which can lead to fatal errors, such as confusing Death Cap mushrooms with edible ones. Additionally, delayed symptom onset complicates timely medical treatment.

Local mushroom is a mushroom that is widely used as food in different parts of the world, especially in hilly areas with humid climate. Wild-harvested mushrooms can be fatal when consumed due to local communities' inability to distinguish poisonous from non-poisonous varieties [6]. Mushroom poisoning symptoms are categorized as early, appearing within 1 to 6 hours and including nausea, vomiting, and diarrhea. Late symptoms arise after 24 hours and involve severe complications like renal failure, seizures, and jaundice [6]. Approximately 140 000 species of fungi have been identified in the world. About 2700 species are considered safe for humans and have nutritional value and medicinal properties. 100 species are considered poisonous to humans [7]. There is a total of 50 species of poisonous mushrooms identified in Iran. At least three species (Kermanshah province, Ilam), Lepiota brunneioncarnata, Hypholoma fascicalare, and Coprinopsis atramentaria (Kohgiloyeh and Boyer Ahmad) are involved in this fungus. The mushroom Lepiota brunneioncarnata with gastrointestinal symptoms 6 to 10 hours after consumption, containing amounts of the toxic substance alpha-ammunition, has irreversible toxic effects on the liver and kidneys and is usually fatal [7]. Atramentaria is a poisonous mushroom with low casualties. This type of mushroom contains cuprin, a protoxin that inhibits acetaldehyde hydrogenase and produces a disulfiram-like reaction if ethanol is consumed within 30 minutes to 3 days after consuming the mushroom. Clinical manifestations after mushroom consumption include gastrointestinal (nausea, vomiting, abdominal pain), nervous (headache, dizziness) and cardiac (tachycardia, hypotension, palpitations) symptoms. Hot flashes, diaphoresis, and shortness of breath [7]. This is the same as our case.

In case report study that published by samy karahan his colleagues, they reported several cases of pancreatitis induced mushroom poisoning [8]. A wide variety of cardiac manifestations were reported, ranging from a simple elevation of cardiac enzymes to ventricular tachycardia, acute heart failure and myocarditis [4]. Avoid any wild and unfamiliar mushrooms to avoid poisoning. 2- Only use mushrooms that are packaged and licensed by the Ministry of Health. 3- If you see signs of poisoning, go to the medical center immediately.

## CRediT statement

Dr. Mehsa Rizebandi and Dr. Faezeh Sehatpour examined and monitored the patient and also reviewed sonography and tests. Mr. Farhad Mohammadi helped in developing the article and writing and analyzing it.
